# Surveillance After Focal Therapy for Prostate Cancer: A Comprehensive Review

**DOI:** 10.3390/cancers17081337

**Published:** 2025-04-16

**Authors:** Jason Koehler, Simon Han, Samuel Tremblay, Wei-Wen Hsu, Bora Kalaycioglu, Aytekin Oto, Abhinav Sidana

**Affiliations:** 1 College of Medicine, University of Cincinnati, Cincinnati, OH 45267, USA; 2 Pritzker School of Medicine, University of Chicago, Chicago, IL 60637, USA; 3 Section of Urology, Department of Surgery, University of Chicago, Chicago, IL 60637, USA; samuel.tremblay@uchicagomedicine.org; 4 Division of Biostatistics and Bioinformatics, College of Medicine, University of Cincinnati, Cincinnati, OH 45267, USA; 5 Department of Radiology, University of Chicago, Chicago, IL 60637, USA

**Keywords:** prostate cancer, focal therapy, surveillance, failure

## Abstract

Focal Therapy (FT) is an emerging treatment modality for prostate cancer (PCa). Due to its novelty, the research exploring how patients should be followed-up after treatment is limited. There is currently no established role for non-prostate-specific-antigen (PSA) biomarkers and PSMA PET. However, a combination of PSA testing, multiparametric magnetic resonance imaging (mpMRI), and systematic and targeted biopsies should routinely be used for surveillance after FT. PSA values that rise 1.0 ng/mL over the nadir after twelve months or rise 1.5 ng/mL over nadir after twenty-four to thirty-six months should raise suspicion for recurrence. The standard imaging technique is mpMRI, but it can often be difficult to interpret after FT, so using a scoring system such as prostate imaging after focal ablation (PI-FAB) or the transatlantic recommendations for prostate gland evaluation with magnetic resonance imaging after focal therapy (TARGET) allows for greater consistency between readers. This review seeks to summarize the current literature regarding surveillance after FT as it relates to biomarkers, imaging, biopsies, and consensus statements.

## Simple Summary

Focal therapy for prostate cancer is a newer way to treat prostate cancer that does not involve surgery to remove the prostate or radiation. However, because it is a more recent form of treatment, little is known regarding monitoring patients after focal therapy to make sure that any cancer recurrence is discovered quickly. This article seeks to review the current research concerning follow-up for patients after focal therapy. Our review highlights the importance of regular surveillance consisting of prostate-specific antigen testing, screening magnetic resonance imaging, and scheduled biopsies to ensure early detection of cancer recurrence after the patient has been treated with focal therapy.

## 1. Introduction

Prostate cancer (PCa) is the most common cancer among men in the USA. Historically, the primary treatments for localized PCa were radical prostatectomy (RP) and radiation therapy (RT). However, RP and RT carry significant morbidity for patients. A significant number of patients experience long-term urinary, sexual, and bowel dysfunction following definitive treatment [1].

Focal Therapy (FT) is an emerging alternative treatment option for PCa that is confined to a section of the prostate. Advances in imaging, particularly mpMRI, have made it possible to localize tumors within the prostate [2]. FT uses a wide variety of modalities (high intensity focused ultrasound (HIFU), cryoablation, irreversible electroporation (IRE), etc.) to ablate the cancerous prostatic tissue and spare the rest of the prostate. FT has been shown to carry a favorable side effect profile, preserving urinary and sexual function [3]. It has also been shown to have favorable short-term oncological efficacy [4,5,6]. More trials are currently investigating longer-term oncological outcomes [7].

Surveillance after treatment is a critical aspect of cancer management to monitor for signs of recurrence, metastases, and side effects. However, the surveillance strategy after FT is not well defined and typically involves a combination of periodic PSAs, imaging, and prostate biopsy. Considering a recent survey indicated that almost half of American urologists use FT, this issue has significant clinical implications [8]. This article seeks to review the current literature regarding surveillance tests, highlighting their utility (or lack thereof) after FT.

## 2. Biochemical Markers

### 2.1. Prostate-Specific Antigen

Prostate-specific antigen (PSA) is the mostly widely used biomarker in FT surveillance. However, establishing appropriate threshold values for PSA poses a challenge in surveillance after FT. While the raw PSA value tends to be used in initial PSA screening, more commonly used PSA metrics in post-FT surveillance include the PSA reduction percentage, nadir, density, and velocity.

After treatments such as RP, PSA reduction is more straightforward, as the value should be approaching 0 ng/mL. However, there is significant variation in the size of the ablation zone in FT resulting in variable declines in PSA values after treatment. Therefore, there is currently no consensus on what value defines a biochemical recurrence in FT [9]. Nonetheless, the typical decline can be expected to be over 50% [4,6,10,11]. Some have set the benchmark for a proper ablation at a decrease in PSA of 70% [12]. Two studies have found that a smaller PSA reduction is a predictor of the need for additional treatments after FT, but one of these studies found it is not an indicator of treatment failure [13,14]. Therefore, it appears that a small PSA reduction may cause preemptive salvage treatment before the patient has treatment failure.

The lowest PSA value after treatment is referred to as the PSA nadir. Typically, the PSA nadir is reached around 6 months after FT [15]. While the PSA nadir varies by patient, the median value is approximately 1–3 ng/mL [15,16,17,18,19]. Some studies have shown a significant correlation between the nadir PSA value and recurrence while others have indicated no significant correlation with treatment failure [14,17,18] Rather than using the nadir value itself, a more reliable predictor of PCa recurrence after FT is the use of a specific increase over the nadir value. Huber et al. analyzed almost 600 FT patients to identify the most accurate PSA nadir values for predicting recurrence [20]. They found that an increase of 1.0 ng/mL over PSA nadir at 12 months or 1.5 ng/mL over nadir at 24 months were the most accurate predictors of recurrence. These criteria showed a sensitivity of 73.5% and 100%, specificity of 70.7% and 71.6%, and a negative predictive value (NPV) of 96.1% and 100%, respectively [20]. In a validation study, meeting one of these criteria was more accurate at predicting recurrence than the PSA nadir value, time to reach PSA nadir, and the percentage of PSA reduction [14].

Another PSA derivative which is studied in FT surveillance is PSA density. The PSA density is calculated by dividing the PSA value by the volume of the prostate. Some correlation does seem to exist between PSA density after FT and the likelihood of recurrence, though it is a weak predictor based on a receiver operating characteristic (ROC) score of 0.62 [17]. Two studies have shown conflicting results for whether there is a significant difference in recurrence rates after FT based on pretreatment PSA density [10,18].

Similar to PSA density, the role for PSA velocity after FT is not well established. PSA velocity describes the rate at which the PSA value is rising, typically described as the change per year. Nguyen et al. indicated that adding the criteria of a PSA velocity of 0.75 ng/mL/year with the criteria of 2 ng/mL over PSA nadir significantly increased the diagnostic yield for recurrence in focal brachytherapy [21]. King et al. discovered that a PSA velocity of 3.0 ng/mL/year or greater after focal brachytherapy is associated with a significant increase in rates of metastasis and PCa-specific mortality. However, velocities of 0.75 ng/mL/year and 1.5 ng/mL/year are not associated with these increases [22]. Kongnyuy et al. demonstrated that PSA velocity is significantly associated with biochemical recurrence (defined as nadir + 2 ng/mL), while doubling time is not [10]. Overall, PSA certainly plays a role in surveillance after FT, but the specific parameters for suspecting recurrence are an active research area. The current research suggests that an increase of 1.0 ng/mL over PSA nadir at 12 months or 1.5 ng/mL over nadir at 24 months may be the most accurate PSA-based predictors for PCa recurrence after FT. Detailed performance characteristics for different PSA derivatives are detailed in Table 1.

### 2.2. Non-PSA Biomarkers

The use of non-PSA liquid biomarkers to monitor oncological control after FT is an emerging area of research, in an effort to reduce the reliance on invasive biopsies. While several biomarkers are available for PCa detection, none have been validated specifically for surveillance after FT. A 2022 Delphi Consensus from the Focal Therapy Society explored potential biomarkers for this setting and found little to no evidence supporting the use of non-PSA biomarkers in the post-FT setting [23]. Notably, the Consensus specifically recommended against the use of prostate cancer antigen 3 (PCA3) in the context of surveillance after FT due to its relatively poor diagnostic accuracy. A recent systematic review and meta-analysis by Kawada et al. examined 49 studies on several liquid biomarkers and their associated tests, including PCA3, SelectMDx, Prostate Health Index (PHI), 4Kscore, MyProstateScore, and ExoDx Prostate. They confirmed that PCA3 offers the lowest diagnostic accuracy among the biomarkers assessed [24]. In the same study, the authors found that among the biomarkers analyzed, other than PCA3, all offered comparable pooled accuracies with 4Kscore offering the highest sensitivity and specificity at 0.87 and 0.58, respectively [24]. Nonetheless, the Delphi Consensus found insufficient evidence to recommend the use of any alternative liquid biomarker tests, reflecting the paucity of data to support the use of non-PSA biomarkers in surveillance after FT in clinical context.

It is critical to note that these biomarkers were developed for diagnostic purposes in treatment-naive patients. While some biomarkers, like PHI, have demonstrated utility in monitoring patients after traditional treatments like radical prostatectomy, the distinct therapeutic approach of FT limits the application of this data for patients who have undergone FT [25]. Thus, further long-term research is needed to elucidate the role of non-PSA biomarkers in prostate cancer surveillance after FT.

## 3. Imaging

### 3.1. Multiparametric MRI

One of the most helpful tools for evaluation of PCa is multiparametric magnetic resonance imaging (mpMRI). Due to its efficacy, it has quickly become the gold standard imaging technique for PCa detection. It has been shown to have a high sensitivity and moderate specificity for identifying PCa in treatment-naïve patients [26]. Its efficacy in detecting recurrence after FT is also established. A systematic review and meta-analysis by Ahn et al. indicated a sensitivity of 0.69 and a specificity of 0.88 for mpMRI after partial gland HIFU [27]. Another systematic review and meta-analysis by Séguier et al. indicated a pooled sensitivity of 0.52, specificity of 0.81, NPV of 0.82, and PPV of 0.50 for mpMRI after focal HIFU [28]. A recent study of 142 patients after FT found mpMRIs read by three readers to have a wide range of sensitivity from 0.33 to 0.73, specificity from 0.57 to 0.97, NPV from 0.93 to 0.96, and PPV from 0.17 to 0.56 [29]. A possible reason for such large variability when reading mpMRIs after FT is the lack of a standard grading system.

The Prostate Imaging–Reporting and Data System (PI-RADS) grading system was designed for treatment-naïve tissue, so it is difficult to apply to ablation zones due to treatment-related changes [30,31]. Therefore, systems for reporting MRI results specifically after FT are in the process of development. The prostate imaging after focal ablation (PI-FAB) three-point scoring system was proposed by Giganti et al. in 2023 and has since been shown to have mixed results in two studies [32]. One study demonstrated a moderate specificity of 54–62%, a high sensitivity of 93%, and an accuracy of 71% [33]. However, the other study found a high specificity of 87–98% and a lower sensitivity of 14–43% [34]. Nonetheless, both studies concluded that the inter reader agreement is moderate to high [33,34]. Another proposal emerged in 2024 with the transatlantic recommendations for prostate gland evaluation with magnetic resonance imaging after focal therapy (TARGET) [35]. These recommendations include timing of the mpMRI, technical aspects, as well as interpretation. Timing of the mpMRI is important, as the prostate undergoes changes in appearance over time after FT, which may affect the diagnostic ability of mpMRI [36]. They propose a five-point scoring system for grading the suspicion of PCa. Due to the recent development of this system, there is only one study that has compared PI-FAB with TARGET [37]. The study indicated that the systems have comparable agreement levels and PI-FAB has a slight advantage in sensitivity (93% in PI-FAB vs. 79–93% in TARGET) and TARGET has the advantage in specificity (54–62% in PI-FAB vs. 62–79% in TARGET) [37]. Using either PI-FAB or TARGET scoring systems creates a standardized score for reading mpMRI images after FT, allowing for high consistency between readers. These scoring systems are promising, and further research involving larger cohorts is needed to validate their utility.

### 3.2. PSMA PET

Prostate-specific membrane antigen positron emission tomography (PSMA PET) has recently emerged as a useful imaging tool for detecting PCa. It is particularly useful when combined with a CT or MRI. PSMA PET/CT has been shown to have better accuracy in detecting nodal and distant metastatic PCa compared to a conventional CT scan and bone scan [38,39,40]. Additionally, a systematic review by Manfredi et al. indicated that PSMA PET/MRI exhibits better accuracy in detecting primary PCa compared to mpMRI [41]. Other studies have indicated that lesion intensity on PSMA PET/CT prior to treatment may have prognostic significance [42,43]. PSMA PET/MRI and PSMA PET/CT have shown a promising ability to detect recurrence after PCa treatment [44,45].

Due to the novelty of both PSMA PET and FT, there is limited research exploring the use of PSMA PET for surveillance specifically after FT. One small pilot study with a short follow-up showed that PSMA PET/MRI and mpMRI both identified the one patient with clinically significant residual PCa after focal HIFU [46]. Another study demonstrated that in a small cohort of patients post-focal HIFU with a negative mpMRI but positive surveillance biopsy, PET/MRI demonstrated a sensitivity, specificity, positive predictive value (PPV), and NPV of 55%, 100%, 100%, and 85%, respectively [47]. Of the ten patients, PSMA PET/MRI discovered all six patients with Gleason score (GS) 4 + 3 lesions [47]. Additionally, they found that PSMA PET/MRI was negative for all four patients with GS 3 + 4 lesions [47]. Another study of one hundred patients post FT indicated that PET/CT has a sensitivity, specificity, PPV, and NPV of 94%, 25%, 91%, and 33%, respectively [48]. While the current limited data is promising, larger, longer-term studies are needed to further evaluate the accuracy and utility of PSMA PET for surveillance after FT. Full performance characteristics for different imaging modalities are detailed in Table 2.

## 4. Biopsies

Due to limitations in the accuracy of biochemical markers as well as imaging techniques in assessing for oncological control after FT, histopathology remains the gold standard for detecting residual or recurrent cancer after FT [49,50,51]. However, the optimal approach to biopsy continues to be a subject of debate. Specifically, questions arise when considering when to conduct biopsies (for-cause vs. protocol) and the biopsy template (targeted vs. systematic).

A “for-cause” biopsy approach typically involves performing biopsies only in cases of a biochemical recurrence or if suspicious lesions are identified on imaging. In contrast, a “for-protocol” approach mandates biopsies regardless of changes in PSA levels or imaging findings. Short term data within 1 year suggests similar tumor-free survival (TFS) rates between these two methods. Stabile et al. demonstrated a 94% TFS rate using a for-cause biopsy protocol, whereas Ladjervardi et al. reported a 86.6% TFS rate [15,52]. This difference is likely attributable to lower sensitivities of both biochemical and imaging parameters, as protocol biopsies are more likely to identify residual or recurrent disease that is not apparent through other methods of monitoring. In the medium term (1–3 years post-FT), studies using a for-cause biopsy approach show TFS rates of 84–87% while for-protocol biopsies exhibit broader ranges of TFS, from 76.2 to 91% [11,52,53,54,55,56,57]. The relatively modest differences indicate that both approaches offer comparable rates of identifying PCa after treatment. However, some have noted that “for-cause” biopsies might be best performed only in centers with extensive experience with FT [58]. Since there were significant heterogeneities in these studies (disease status, the approach of FT, surgeon experience, etc.) no consistent conclusion can be drawn, further underscoring the need for larger and longer studies to better understand these trends.

An important consideration in biopsy techniques is whether to conduct targeted biopsies, systematic biopsies, or a combination of both. Targeted biopsies focus solely on the ablation zone or a suspicious lesion, while systematic biopsies sample the entire prostate, including areas outside the ablation zone, which are often still under active surveillance. Several studies highlight the critical nature of systematic biopsies, as out-of-field recurrence (OFR) rates are comparable to, and in some cases may exceed, in-field recurrence (IFR) rates. Sidana et al. found that in 101 men who underwent biopsy after FT, 8.9% had IFR while 7.9% had OFR at a median follow-up of 33 months [6]. Similarly, a study by Tourinho-Barbosa et al. examined outcomes after FT in 309 men (treated with either HIFU or IRE) with a median follow-up of 45 months and found that 33% experienced IFR and 19% had OFR [18]. Other studies have shown that the rates of OFR may even exceed IFR, with overall OFR rates of 13% to 16%, compared to IFR rates of 3% to 7% [55,59]. These findings emphasize the importance of performing systematic biopsies to ensure comprehensive monitoring of all cases of clinically significant PCa after FT.

## 5. Guidelines and Consensus Statements

While individual studies are helpful for advancing understanding of a field, guidelines and consensus statements are often the most influential items in affecting how clinicians practice. The guidelines from the American Urological Association regarding follow-up after FT are unclear. The expert opinion states that patients after FT, “should be followed post-ablation with PSA, digital rectal exam (DRE), MRI, and biopsy tailored to their specific health and cancer characteristics” [60]. The European Association of Urology recommends FT only in the context of a clinical trial or registry and offers no instruction for surveillance after FT [61]. These guidelines leave clinicians with little guidance in how and when to use the various tools, such as a PSA, DRE, MRI, and biopsy for their patients that have undergone FT.

Compared to the above guidelines, more tangible instructions are offered in expert consensus statements. In a consensus statement by Borkowetz et al., ninety-five percent of the experts agreed that a targeted and systematic biopsy should be obtained 6–12 months after FT [62]. Another consensus statement agrees that a protocol repeat biopsy should be performed, as well as a screening mpMRI [63]. The most detailed consensus statements were conducted by Lebastchi et al. and Muller et al. among experts on surveillance after FT [49,51]. Consensus was achieved with the agreement of at least 80% of the experts. The use of a PSA, MRI, systematic and targeted biopsies, and functional outcome measurements after FT attained consensus. The consensus statement by Lebatschi et al. recommends that PSAs should be collected every three months for the first year then every six months. It recommends an MRI at the 6-month and 18-month mark. Also, it calls for a “for-protocol” systematic and targeted biopsy at 6–12 months. Additionally, functional outcome monitoring should be included at 3–6 months and beyond until stability is attained. The consensus statement by Muller et al. is very similar. However, the two consensus statements disagree primarily on imaging. Muller et al. recommend six surveillance mpMRIs for follow-up while Lebastchi et al. only recommend two mpMRIs [49,51]. However, regardless of which consensus statement is followed, these statements give practitioners tangible guidance for routine surveillance for their FT patients.

The authors adhere to the following protocol in their institution. A PSA is obtained at three and six months and then every six months. The Sexual Health Inventory for Men score and the International Prostate Symptom Score are obtained at three, six, and twelve months after FT. At six to twelve months after FT, the patient undergoes a mpMRI followed by a protocol systematic and targeted biopsy of the ablation zone along with any new suspicious lesions. If the biopsy is positive for recurrence, the patient is managed according to the risk stratification and patient preferences. If the biopsy is negative, the patient repeats mpMRIs at two- and four-years post ablation with further “for cause” biopsies if needed. Overall, guidelines, consensus statements, and the authors’ own experience confirm the necessity of using a PSA, mpMRI, and biopsies for surveillance in patients after FT.

## 6. Discussion

As FT for the treatment of localized PCa has increased in popularity, the necessity for standardized follow-up protocols has emerged. Based on the current literature, a PSA, mpMRI, and systematic and targeted biopsies are essential aspects of surveillance after FT. The use of other biomarkers and PSMA PET has little research yet to support their use. The level of PSA rise to warrant ordering an mpMRI is reliant on the clinical decision making of the provider, but the current proposed criteria by Huber et al. of an increase of 1.0 ng/mL over PSA nadir at 12 months or 1.5 ng/mL over nadir at 24 months give an indication for when to suspect recurrence. Several routine mpMRIs should also be performed and read by a radiologist experienced in interpreting prostates after FT. Based on the recent developments in scoring systems for mpMRI, the research has yet to establish whether PI-FAB or TARGET is superior. As a biopsy is the only definitive way to diagnose PCa, one routine systematic and targeted biopsy should be performed on every patient after FT, and it should also be performed when there is suspicion of recurrence.

Many gaps remain in the research regarding follow-up after FT. Specifically, there is a lack of long-term outcome trials exploring the most effective follow-up regimen after FT. Additionally, more research is needed to definitively identify specific values for PSA that can be used as cutoffs for suspecting recurrence. PSMA PET has very promising initial studies, but it still requires further study to fully elucidate its role in surveillance after FT. Another promising area needing further exploration is the combination of various factors to influence the need for a biopsy, as a recent study indicated the integration of PSA and mpMRI findings may reduce the need for a surveillance biopsy [64]. Because most of the current literature evaluates HIFU, any need for different follow-up protocols for different modalities of FT needs further investigation. As these areas are explored, an evidence-based approach for surveillance for patients after FT will emerge.

## 7. Conclusions

Close monitoring is required after patients undergo FT to ensure that any PCa recurrence is quickly identified. Currently, clinicians using FT have limited guidance on how to appropriately follow their patients after the procedure. Regular PSA levels, several mpMRIs, and systematic and targeted biopsies should be included in the surveillance for patients after FT for the treatment of PCa. The use of alternative biomarkers and PSMA PET is currently not supported based on the current literature. Overall, surveillance after FT uses well established tools for detecting recurrent PCa, but the evidence regarding when and how the tools should be used needs further elucidation.

## Figures and Tables

**Table 1:** Performance characteristics of various PSA derivatives for identifying recurrent PCa after FT.

Derivative	Sensitivity	Specificity	PPV	NPV	HR	ROC	Source(s)
% PSA reduction	N/A	N/A	N/A	N/A	0.97 (0.96–0.98) *	N/A	Mattlet et al. ^b^ [14]
PSA nadir	N/A	N/A	N/A	N/A	1.23 (1.08–1.41) *	0.58	Dickinson et al. ^c^, Mattlet et al. ^b^ [14,17]
PSA of 1.0 ng/mL over nadir at 12 months	73%	71%	N/A	96%	10 (4.52–22.1) ^a^	N/A	Huber et al. ^d^, Mattlet et al. ^b^ [14,20]
PSA of 1.5 ng.ml over nadir at 24 months	100%	72%	N/A	100%	10 (4.52–22.1) ^a^	N/A	Huber et al. ^d^, Mattlet et al. ^b^ [14,20]
PSA density after FT	N/A	N/A	N/A	N/A	N/A	0.62	Dickinson et al. ^c^ [17]

* Statistically significant result (*p* < 0.05). ^a^ HR for one or the other criteria. ^b^ 2023 retrospective review of 178 HIFU patients. Recurrence defined as clinically significant PCa on postoperative biopsy, the need for salvage radical or systematic treatment, metastasis, or PCa-related death. ^c^ 2017 prospective study of 118 HIFU patients. Recurrence defined by positive biopsy. ^d^ 2020 prospective study of 598 HIFU patients. Recurrence defined as any secondary treatment, tumor recurrence with Gleason 3 + 4 or greater disease on prostate biopsy, metastasis, or PCa-related mortality. HR: Hazard ratio.

**Table 2:** Performance characteristics of various imaging modalities for identifying recurrent PCa after FT.

Modality	Sensitivity	Specificity	PPV	NPV	ROC	Source(s)
MRI	81%	91%	N/A	N/A	0.81	Ahn et al. ^a^ [27]
MRI	52%	81%	50%	82%	N/A	Séguier et al. ^b^ [28]
MRI within 3 weeks of FT	68%	59%	47%	77%	0.69	Dickinson et al. ^c^ [17]
MRI at median of 6 months	60%	78%	62%	77%	0.76	Dickinson et al. ^c^ [17]
MRI PIFAB	93%	58%	57%	93%	0.75	Esengur et al. ^d^ [36]
MRI TARGET	86%	71%	64%	90%	0.79	Esengur et al. ^d^ [36]
PSMA PET/CT	94%	25%	91%	33%	N/A	Jafarvand et al. ^e^ [47]
PSMA PET/MRI after positive biopsy with negative MRI	55%	100%	100%	85%	N/A	Burger et al. ^f^ [46]

^a^ 2022 meta-analysis of 19 studies: 703 HIFU patients. No standard definition of recurrence. ^b^ 2024 meta-analysis of 12 studies: 1046 HIFU patients. Recurrence defined by positive biopsy. ^c^ 2017 prospective study of 118 HIFU patients. Recurrence defined by positive biopsy. ^d^ 2025 retrospective review of 28 focal laser ablation, six HIFU, and four cryoablation patients. Recurrence defined by positive biopsy. ^e^ 2023 retrospective study of 100 FT patients, mostly HIFU and cryoablation. Recurrence defined by positive biopsy within 3 months of PET scan. ^f^ 2019 prospective review of 10 HIFU patients. Recurrence defined by positive biopsy.

## Abbreviations

The following abbreviations are used in this manuscript:

DRE	Digital rectal exam
FT	Focal therapy
HIFU	High intensity focused ultrasound
HR	Hazard ratio
IFR	In-field recurrence
IRE	Irreversible electroporation
mpMRI	multiparametric MRI
NPV	Negative predictive value
OFR	Out of field recurrence
PCa	Prostate cancer
PCA3	Prostate cancer antigen 3
PHI	Prostate health index
PI-FAB	Prostate imaging after focal ablation
PI-RADS	Prostate Imaging–Reporting and Data System
PPV	Positive predictive value
PSA	Prostate specific antigen
PSMA PET	Prostate specific membrane antigen positron emission tomography
ROC	Receiver operating characteristic
RP	Radical prostatectomy
RT	Radiation therapy
TARGET	Transatlantic recommendations for prostate gland evaluation with magnetic resonance imaging after focal therapy
TFS	Tumor free survival
